# Public Health and Hygiene

**Published:** 1898-01

**Authors:** 


### Public Health and Hygiene.

---

Buffalo continues to lead the list as the healthiest city of its class in the world. The reports for 1897 that are just now tabulating at the health office show the death-rate to have been 12.42 per

1,000 inhabitants, as against 12.72 for 1896. The total number of deaths for 1897 is given at 4,472, of which 2,266 were males and 2,206 females. The estimated population is 360,000.

In 1891 there were 6,001 deaths, an increase of 1,529 over 1897. The population in that year was estimated at 255,000.

Last year there were 8,983 births, of which 4,511 were males and 4,472 females. This is an increase of 569 over 1896, when the births were 8,414. There were 2,542 marriages last year as against 2,525 in 1896.

One of the most notable facts in connection with the record last year was the decrease in the number of deaths from cholera infantum. In 1896, 371 babies lost their lives from that disease. Last year the number was 277.

Dr. Wende figures that the good health of the people of Buffalo is largely due to the improvements made in the sanitary arrangements in the houses, to the excellent way his subordinates do their work and the strict enforcement of the health ordinances.

